# Clinical Efficacy and Adverse Effects of Antibiotics Used to Treat *Mycobacterium abscessus* Pulmonary Disease

**DOI:** 10.3389/fmicb.2019.01977

**Published:** 2019-08-23

**Authors:** Jianhui Chen, Lan Zhao, Yanhua Mao, Meiping Ye, Qi Guo, Yongjie Zhang, Liyun Xu, Zhemin Zhang, Bing Li, Haiqing Chu

**Affiliations:** ^1^Department of Respiratory Medicine, Shanghai Pulmonary Hospital, Tongji University School of Medicine, Shanghai, China; ^2^Tongji University School of Medicine, Shanghai, China; ^3^Shanghai Key Laboratory of Tuberculosis, Shanghai Pulmonary Hospital, Tongji University School of Medicine, Shanghai, China

**Keywords:** *Mycobacterium abscessus*, pulmonary disease, drug, efficacy, adverse effect

## Abstract

Treatment of *Mycobacterium abscessus* pulmonary infection requires long-term administration of multiple antibiotics. Little is known, however, about the impact of each antibiotic on treatment outcomes. A retrospective analysis was conducted to evaluate the efficacy and adverse effects of antibiotics administered in 244 cases of *M. abscessus* pulmonary disease. Only 110 (45.1%) patients met the criteria for treatment success. The efficacy of treating *M. abscessus* pulmonary disease continues to be unsatisfactory especially for infections involving *M. abscessus* subsp. *abscessus*. Treatment with drug combinations that included amikacin [adjusted odds ratio (AOR), 3.275; 95% confidence interval (CI), 1.221–8.788], imipenem (AOR, 2.078; 95% CI, 1.151–3.753), linezolid (AOR, 2.231; 95% CI, 1.078–4.616), or tigecycline (AOR, 2.040; 95% CI, 1.079–3.857) was successful. Adverse side effects affected the majority of patients (192/244, 78.7%). Severe effects that resulted in treatment modification included: gastrointestinal distress (29/60, 48.3%) mostly caused by tigecycline, ototoxicity (14/60, 23.3%) caused by amikacin; and myelosuppression (6/60, 10%) caused mainly by linezolid. In conclusion, the success rate of treatment of *M. abscessus* pulmonary disease is still unsatisfactory. The administration of amikacin, imipenem, linezolid, and tigecycline correlated with increased treatment success. Adverse side effects are common due to long-term, combination antibiotic therapy. Ototoxicity, gastrointestinal distress, and myelosuppression are the most severe.

## Introduction

The incidence of pulmonary infections caused by non-tuberculous mycobacteria (NTM) has increased dramatically worldwide in recent years (Hoefsloot et al., 2013; Lin et al., 2018; Lee et al., 2019). Among them, *Mycobacterium abscessus* (*M. abscessus*) infections are the most difficult to manage (Nessar et al., 2012; Griffith, 2019). *M. abscessus* infections, which are even refractory to combined, long-term antibiotic therapy, often result in mortality.

*Mycobacterium abscessus* treatment is challenging, albeit effective treatment options are evolving. In 2007, the American Thoracic Society (ATS)/Infectious Disease Society of America (IDSA) introduced a clarithromycin-based multidrug therapy with amikacin plus cefoxitin or imipenem administered parenterally (Griffith et al., 2007). In 2017, the British Thoracic Society guidelines recommended a revision in antibiotic therapy that consisted of intravenous amikacin, tigecycline, and imipenem with a macrolide, e.g., clarithromycin, for the initial treatment phase (Haworth et al., 2017). This was followed by a continuation phase composed of nebulized amikacin and a macrolide in combination with additional oral antibiotics. It was further recommended that selection of a specific agent should consider the antibiotic susceptibility of the isolate and the antibiotic tolerance of the patient.

Patients with pulmonary disease due to *M. abscessus* infection require long-term treatment with multiple antibiotics. Little is known about the impact of each antibiotic on treatment outcomes. Recently, the NTM International Network released a consensus statement defining the treatment outcomes of NTM pulmonary disease, allowing for a better evaluation of the efficacy of each antibiotic used in clinical studies (van Ingen et al., 2018). Using these criteria, Kwak et al. (2019) conducted an excellent meta-analysis of 14 studies with detailed individual patient data. Patients treated with drug combinations that included azithromycin, amikacin, or imipenem exhibited better outcomes, emphasizing the import of different therapeutic approaches. However, two important antibiotics specifically recommended in the 2017 British Thoracic Society guidelines, i.e., linezolid and tigecycline, were not used or were administered in very few cases. Moreover, despite identifying the antibiotics most effective, the adverse effects of these antibiotics were not considered.

We previously reported a series of studies demonstrating the antibiotic susceptibility of clinical *M. abscessus* isolates and the treatment outcomes of patients diagnosed with *M. abscessus* pulmonary disease (Li B. et al., 2017, 2018; Guo et al., 2018; Ye et al., 2019). A number of cases accumulated during the course of these studies dealt with the long-term treatment with antibiotics, including linezolid and tigecycline; the adverse effects of antibiotic treatment were well documented. The retrospective analysis reported herein was undertaken to evaluate the efficacy and adverse effect of a variety of antibiotics used to treat *M. abscessus* pulmonary disease. The results of this analysis should facilitate therapeutic choices in clinical practice.

## Materials and Methods

### Study Population

**FIGURE 1:**
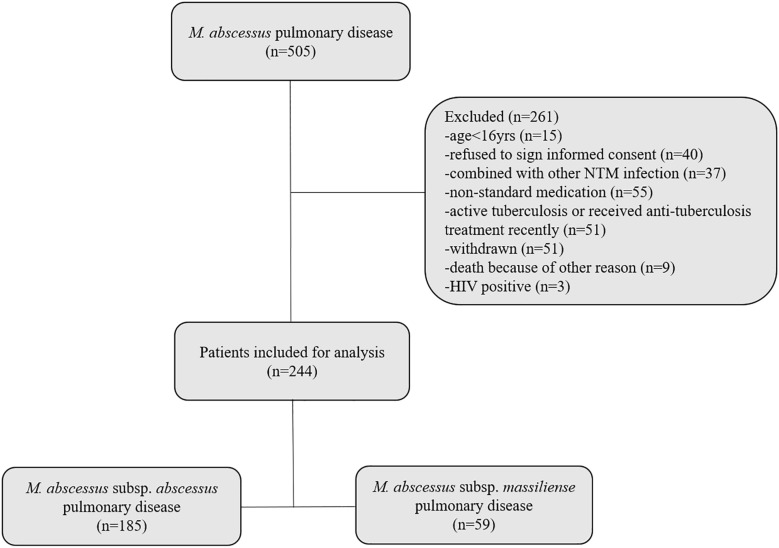
Flow diagram of the study. Two hundred forty-four pulmonary disease patients, who conformed to the inclusion criteria, were enrolled. One hundred eighty-five patients were infected with *M. abscessus* subsp. *abscessus*; 59 patients were infected with *M. abscessus* subsp. *massiliense*.

A retrospective review was conducted of the medical records of all patients entering Shanghai Pulmonary Hospital between January 2012 and December 2017 with *M. abscessus* lung disease. Participating patients were followed-up on a regular basis; sputum culture and chest CT examination were performed once a month and once every 3 months, respectively. The inclusion criteria were: (1) age > 16 years; (2) having undergone initial diagnosis and treatment at the Shanghai Pulmonary Hospital in accordance with the 2007 ATS/IDSA Guidelines or the 2017 British Thoracic Society Guidelines; and (3) follow-up period lasting >12 months. Exclusion criteria were: (1) age < 16 years; (2) co-infection with active tuberculosis or another NTM; (3) refusal to sign informed consent form; and (4) AIDS. Notably, patients with cystic fibrosis were never found and are essentially non-existent in Asia. A detailed, patient enrollment flow chart is shown in Figure 1. This study was approved by the Ethics Committees of Shanghai Pulmonary Hospital and Tongji University School of Medicine, ethics number K17-150. All participants signed informed consent forms before enrollment.

### Collection, Identification, and Preservation of Bacteria

All clinical *M. abscessus* isolates used in this study were preserved in the Clinical Microbiology Laboratory of Shanghai Pulmonary Hospital. Shanghai Pulmonary Hospital is one of the designated treatment centers for tuberculosis and NTM in China, attracting NTM cases nationwide. *M. abscessus* isolates were obtained from sputum and bronchoalveolar lavage fluid. The detailed process of *M. abscessus* identification was described previously by us using *rpoB*, *erm*(41), and *PRA-hsp65* genes to identify and differentiate *abscessus*, *massiliense*, and *bolletii* subspecies (Guo et al., 2018). *M. abscessus* subsp. *bolletii* is extremely rare and, therefore, was excluded. Identified isolates, stored at −80°C, were recovered for microbiology and molecular biology studies.

### Genotype Analysis

Genomic information of *rpoB*, *erm*(41), and *PRA-hsp65* genes for 182 isolates was obtained by whole genome sequencing, which was available at DDBJ/ENA/GenBank under the BioProject PRJNA448987, PRJNA398137, and PRJNA488058. The genotype of the remaining isolates was determined by PCR and sequencing the *rpoB*, *erm*(41), and *PRA-hsp65* genes.

### Treatment Regimen

All patients were treated with antibiotics recommended by the ATS/IDSA or the British Thoracic Society guidelines (Griffith et al., 2007; Haworth et al., 2017). Clarithromycin, azithromycin, amikacin, tigecycline, linezolid, imipenem, meropenem, cefoxitin, ciprofloxacin, moxifloxacin, doxycycline, minocycline, and levofloxacin (among the most common antibiotics used to treat *M. abscessus* infections) were included in the analysis. These antibiotics were selected based upon: drug susceptibility, adverse side effects, medical history, economic considerations, and the ease with which the regimens could be modified during the course of treatment.

### Treatment Efficacy and Adverse Drug Effects

Treatment outcomes were defined in accordance with the NTM International Network consensus statement (van Ingen et al., 2018); a microbiological cure was considered successful treatment. Since all patients enrolled in the current study were simultaneously or sequentially treated with more than one drug, analysis of the direct response to a single drug was impossible. Rather, the efficacy of individual drugs was assessed based upon a comparison of the frequency of drug usage in successfully versus unsuccessfully treated patients (Kwak et al., 2019). Evaluation of chest images and symptoms was determined by the treating physician. Adverse drug effects and the drugs responsible were identified by referring to the medical records and confirmed by the diminution or elimination of symptoms following drug cessation.

### Statistical Analysis

Statistical analysis was conducted using SPSS version 20 (IBM Corporation, Chicago, IL, United States). Group comparisons for continuous data were performed using Mann–Whitney *U*-test. Group comparisons of proportions were made using Pearson’s chi-squared test or Fisher’s exact test. Multivariable logistic regression was used to confirm the association of specific drug use with treatment success; symptomatic and radiographic improvement; and adjusting for age, sex, BMI, and radiographic features. Statistical significance was set at a two-sided *p*-value of <0.05.

## Results

### Patient Characteristics

Two hundred and forty-four patients who conformed to the recruitment criteria were enrolled. Among them, 75.8% of the patients were infected with *M. abscessus* subsp. *abscessus*; 24.2% were infected with *M. abscessus* subsp. *massiliense* (Table 1). Patients experiencing *M. abscessus* pulmonary disease were 73.0% female and had relatively low body mass indices. Most of the patients had comorbidities consisting of prior TB/NTM infection or bronchiectasis. The main symptoms were cough and sputum production. The proportion of pulmonary disease patients infected with *M. abscessus* subsp. *abscessus* exhibited more fibrocavitary and less nodular bronchiectasis in chest images relative to patients infected with *M. abscessus* subsp. *massiliense*.

**TABLE 1:** Baseline patient characteristics^a^.

	**Total (*n* = 244)**	***M. abscessus* subsp. *abscessus* pulmonary disease (*n* = 185)**	***M. abscessus* subsp. *massiliense* pulmonary disease (*n* = 59)**	***P*-value**
Median age (years)	56.0(49.0,65.8)^b^	56(49.0,66.0)^b^	54.0(48.0,63.0)^b^	0.207
Sex, male	66(27.0)	53(28.6)	13(22.0)	0.319
Body mass index (kg/m^2^)	19.6(18.6,20.5)^b^	19.7(18.6,20.5)^b^	19.4(18.6,20.6)^b^	0.536
**Respiratory comorbidities**				
Prior TB/NTM^c^	127(52.0)	92(49.7)	35(59.3)	0.199
Bronchiectasis	208(85.2)	154(83.2)	54(91.5)	0.118
COPD^c^	16(6.6)	13(7.0)	3(5.1)	0.768
Cor pulmonale	12(4.9)	10(5.4)	2(3.4)	0.736
Asthma	15(6.1)	12(6.5)	3(5.1)	1.000
**Main respiratory symptoms**				
Cough	201(82.4)	153(87.4)	48(81.4)	0.246
Sputum	206(84.4)	158(85.4)	48(81.4)	0.455
Hemoptysis	59(24.2)	47(25.4)	12(20.3)	0.429
Shortness of breath	75(30.7)	54(29.2)	21(35.6)	0.353
Chest pain	48(19.7)	38(20.5)	10(16.9)	0.546
**Radiographic features**				**<0.001**
Fibrocavitary	61(25.0)	57(30.8)	4(6.8)	
Nodular bronchiectatic	171(70.1)	116(62.7)	55(93.2)	
Indeterminate	12(4.9)	12(6.5)	0(0)	

*^*a*^Data are the medians (interquartile range) or numbers (percentage). ^*b*^Range. ^*c*^TB, tuberculosis; NTM, non-tuberculous mycobacterium; COPD, chronic obstructive pulmonary disease.*

### Treatment Outcomes and Modalities

Only 45.1% of total patients (110/244) met the criteria for treatment success (Table 2). Significantly greater success was observed among patients infected with *M. abscessus* subsp. *massiliense* [81.4% (48/59)] compared to those infected with subsp. *abscessus* [33.5% (62/185)]. Clarithromycin used in drug regiments to treat patients infected with *M. abscessus* subsp. *abscessus* was more commonly associated with treatment failure than treatment success (85.4 vs. 71.0%, respectively). Treatments that included a different macrolide (azithromycin), on the other hand, achieved significantly greater success (37.1%) than failure (19.5%). These differences were not found upon analysis of the entire study population or patients infected with *M. abscessus* subsp. *massiliense*. Treatment with drug combinations that included amikacin, imipenem, linezolid, or tigecycline also exhibited far greater success than failure in treating the entire patient population, as well as treating those patients infected with *M. abscessus* subsp. *abscessus*. Drug combinations that included these same four antibiotics did not exert the same beneficial effects on patients infected with *M. abscessus* subsp. *massiliense*.

**TABLE 2:** Comparison of treatment modalities: success versus failure^*a*^.

**Antibiotic**	***M. abscessus* pulmonary disease**				***M. abscessus* subsp. *abscessus* pulmonary disease**				***M. abscessus* subsp. *massiliense* pulmonary disease**			
			
	**Total (*n* = 244)**	**Success (*n* = 110)**	**Failure (*n* = 134)**	***P-*value**	**Total (*n* = 185)**	**Success (*n* = 62)**	**Failure (*n* = 123)**	***P-*value**	**Total (*n* = 59)**	**Success (*n* = 48)**	**Failure (*n* = 11)**	***P-*value**
Clarithromycin	199	86(78.2)	113(84.3)	0.218	149	44(71.0)	105(85.4)	0.020	50	42(87.5)	8(72.7)	0.347
Azithromycin	61	32(29.1)	29(21.6)	0.181	47	23(37.1)	24(19.5)	0.010	14	9(18.8)	5(45.5)	0.110
Amikacin	218	104(94.5)	114(85.1)	0.017	166	60(96.8)	106(86.2)	0.025	52	44(91.7)	8(72.7)	0.112
Imipenem	67	39(35.5)	28(20.9)	0.011	47	22(35.5)	25(20.3)	0.025	20	17(35.4)	3(27.3)	0.734
Meropenem	13	7(6.4)	6(4.5)	0.514	10	5(8.1)	5(4.1)	0.256	3	2(4.2)	1(9.1)	0.468
Cefoxitin	144	65(59.1)	79(59.0)	0.983	110	38(61.3)	72(58.5)	0.719	34	27(56.2)	7(63.6)	0.745
Linezolid^b^	38	24(21.8)	14(10.4)	0.015	27	15(24.2)	12(9.8)	0.009	11	9(18.8)	2(18.2)	1.000
Tigecycline	53	32(29.1)	21(15.7)	0.011	39	19(30.6)	20(16.3)	0.024	14	13(27.1)	1(9.1)	0.269
Doxycycline	30	10(9.1)	20(14.9)	0.167	23	6(9.7)	17(13.8)	0.420	7	4(8.3)	3(27.3)	0.112
Minocycline	22	10(9.1)	12(9.0)	0.971	15	4(6.5)	11(8.9)	0.558	7	6(12.5)	1(9.1)	1.000
Moxifloxacin^b^	53	28(25.5)	25(18.7)	0.200	34	13(21.0)	21(17.1)	0.519	19	15(31.2)	4(36.4)	0.734
Levofloxacin^b^	26	8(7.3)	18(13.4)	0.121	20	4(6.5)	16(13.0)	0.175	6	4(8.3)	2(18.2)	0.310
Ciprofloxacin	17	8(7.3)	9(6.7)	0.865	13	4(6.5)	9(7.3)	1.000	4	4(8.3)	0(0)	1.000
Number of patients administered:	–	–	–	0.810	–	–	–	0.148	–	–	–	0.367
One parenteral drug	15	6(5.5)	9(6.7)	–	9	1(1.6)	8(6.5)	–	6	5(10.4)	1(9.1)	–
Two parenteral drugs	161	70(63.6)	91(67.9)	–	124	38(61.3)	86(69.9)	–	37	32(66.7)	5(45.5)	–
Three parenteral drugs	62	31(28.2)	31(23.1)	–	47	21(33.9)	26(21.1)	–	15	10(20.8)	5(45.5)	–
More than three parenteral drugs	6	3(2.7)	3(2.2)	–	5	2(3.2)	3(2.4)	–	1	1(2.1)	0(0)	–
Months of treatment	25.6 (18.8, 37.8)	20.7	30.0	<0.001	27.7 (20.7, 40.8)	23.4	30.0	0.001	20.2 (15.9, 29.8)	18.0	28.0	0.179
		(16.2, 31.0)	(22.0, 43.3)			(18.1, 34.6)	(22.0, 44.0)			(15.9, 26.8)	(16.0, 43.0)	
Surgical resection	10	2	8	0.192	7	1	6	0.427	3	1	2	0.086

*^*a*^Data are the number (percentage; the number of patients who succeeded or failed treatment with the indicated drug divided by the total number of patients who succeeded or failed treatment) or median (interquartile range) of cases. Each antibiotic listed was included regardless of whether it was discontinued during the course of treatment. ^*b*^Administered orally and/or intravenously.*

The duration of treatment was significantly shorter for the total population of patient who were successfully treated versus patients who failed treatment. Similarly, the treatment duration was substantially shorter for *M. abscessus* subsp. *abscessus* infected patients who were successfully treated. Successfully treated patients infected with *M. abscessus* subsp. *massiliense* exhibited the same trend, but failed to achieve statistical significance. Efficacy of treatment modalities with respect to symptomatic and raidiographic improvement has also been made and similar outcome profiles are obtained (Supplementary Tables 1, 2).

### Effects of Individual Drugs on Treatment Outcomes

Multiple logistic regression analysis (adjusted for age, gender, BMI, and radiographic findings) indicated that azithromycin was clinically superior to clarithromycin in treating patients infected with *M. abscessus* subsp. *abscessus* (Table 3). The superiority of azithromycin was not observed in treating the total patient population or patients infected with *M. abscessus* subsp. *massiliense*. Amikacin, imipenem, linezolid, and tigecycline were also associated with success in treating the entire patient population, as well as those patients infected with *M. abscessus* subsp. *abscessus*. Notably, amikacin was the only drug showing clinical efficacy in treating *M. abscessus* subsp. *massiliense* infected patients in our study. The association of each drug with symptomatic and radiographic improvements was also subjected to multivariable logistic regression analysis (Supplementary Tables 3, 4).

**TABLE 3:** Treatment success with individual antibiotics.

**Antibiotic**	**Total (*n* = 244)**			***M. abscessus* subsp. *abscessus* pulmonary disease (*n* = 185)**			***M. abscessus* subsp. *massiliense* pulmonary disease (*n* = 59)**		
			
	**Adjusted OR^a^**	**95% CI^a,b^**	***P*-value**	**Adjusted OR**	**95% CI**	***P*-value**	**Adjusted OR**	**95% CI**	***P-*value**
Clarithromycin	0.588	0.290–1.194	0.142	0.425	0.191–0.945	0.036	1.460	0.214–9.962	0.699
Azithromycin	1.558	0.844–2.877	0.156	2.339	1.141–4.794	0.020	0.295	0.061–1.418	0.128
Amikacin	3.275	1.221–8.788	0.018	5.911	1.247–28.012	0.025	15.023	1.294–174.400	0.030
Imipenem	2.078	1.151–3.753	0.015	2.050	1.018–4.126	0.044	1.357	0.280–6.575	0.705
Meropenem	1.218	0.390–3.806	0.735	1.787	0.486–6.574	0.382	0.341	0.026–4.487	0.413
Cefoxitin	1.121	0.659–1.908	0.672	1.253	0.656–2.394	0.495	0.610	0.133–2.795	0.524
Linezolid^c^	2.231	1.078–4.616	0.031	2.875	1.221–6.772	0.016	1.286	0.189–8.746	0.797
Tigecycline	2.040	1.079–3.857	0.028	1.971	0.931–4.173	0.076	2.614	0.291–23.514	0.391
Doxycycline	0.599	0.260–1.380	0.229	0.628	0.222–1.772	0.379	0.408	0.053–3.147	0.390
Minocycline	0.992	0.399–2.467	0.986	0.691	0.206–2.315	0.549	1.312	0.116–14.876	0.827
Moxifloxacin^c^	0.695	0.372–1.300	0.255	0.866	0.393–1.908	0.720	1.495	0.303–7.388	0.622
Levofloxacin^c^	0.474	0.193–1.162	0.103	0.453	0.142–1.445	0.181	0.242	0.032–1.857	0.172
Ciprofloxacin	1.026	0.372–2.831	0.960	1.155	0.330–4.039	0.822	0	0	0

*^*a*^OR, odds ratio; CI, confidence interval. ^*b*^Adjusted for age, sex, body mass index, and radiographic findings. ^*c*^Administered orally and/or intravenously.*

### Adverse Effects of Antibiotics

One hundred and ninety-two of the 244 patients enrolled in the study experienced 319 adverse events caused by therapeutic intervention (Table 4). The most frequent adverse events were gastrointestinal complaints that included nausea, vomiting, diarrhea, and abdominal pain. Hematologic toxicity and nephrotoxicity were the next most frequent events documented. Most of these were mild, tolerable, and did not result in disability or death. Serious adverse reactions, however, occurred in 60 (24.6%) patients resulting in a discontinuation or modification of the treatment regimen. Notably, severe myelosuppression was mainly a consequence of linezolid treatment. Gastrointestinal side effects were most often due to tigecycline; amikacin caused most cases of serious ototoxicity and nephrotoxicity. Fortunately, all severe side effects disappeared or were remarkably alleviated after changes in the treatment regimen.

**TABLE 4:** Adverse events^∗^.

			**Antibiotic-specific adverse events leading to treatment modification (*n* = 60)**					
			
	**Total patients (*n* = 192)**	**Total frequency of adverse events (*n* = 319)**	**Clarithromycin (*n* = 4) (199 patients)**	**Azithromycin (*n* = 3) (61 patients)**	**Amikacin (*n* = 26) (218 patients)**	**Imipenem (*n* = 3) (67 patients)**	**Linezolid (*n* = 9) (38 patients)**	**Tigecycline (*n* = 15) (53 patients)**
Gastrointestinal distress	79(41.1)	143(44.8)	4	3	4	0	4	14
Diarrhea	15(7.8)	22(6.9)	0	0	0	0	0	0
Abdominal pain	13(6.8)	25(7.8)	1	1	0	0	1	0
Nausea	35(18.2)	66(20.7)	1	2	4	0	2	10
Vomiting	16(8.3)	30(9.4)	2	0	0	0	1	4
Dizziness	7(2.9)	15(4.7)	0	0	0	0	0	0
Ototoxicity	11(5.7)	15(4.7)	0	0	14	0	0	0
Nephrotoxicity	20(10.4)	34(10.7)	0	0	5	0	0	0
Hepatotoxicity	9(4.7)	15(4.7)	0	0	0	0	0	1
Hematologic toxicity	11(5.7)	26(8.2)	0	0	0	1	5	0
Leukopenia	5(2.6)	11(3.4)	0	0	0	1	2	0
Thrombocytopenia	2(1.0)	5(1.6)	0	0	0	0	2	0
Anemia	4(2.1)	10(3.13)	0	0	0	0	1	0
Insomnia	3(1.6)	6(1.9)	0	0	0	0	0	0
Fever	3(1.6)	5(1.6)	0	0	0	0	0	0
Headache	14(7.3)	22(6.9)	0	0	0	0	0	0
Myoclonus	3(1.6)	4(1.3)	0	0	0	0	0	0
Agitation	3(1.6)	3(0.9)	0	0	0	1	0	0
Taste alteration	10(5.2)	11(3.4)	0	0	0	0	0	0
Allergic reactions	19(9.9)	20(6.3)	0	0	3	1	0	0

*^∗^The patients affected by the adverse event listed are enumerated. Notably, a single patient is often affected by more than one event. Those antibiotics, which most frequently cause events that necessitates treatment modification, are listed.*

## Discussion

The study reported here evaluated the efficacy and adverse effects of different antibiotics used in combination to treat patients with pulmonary disease caused by *M. abscessus*. A variety of antibiotics recommended by the British Thoracic Society guidelines were analyzed including linezolid and tigecycline, two important drugs recently used more frequently. While the overall rate of treatment success remained very low, the incorporation of amikacin, imipenem, linezolid, and/or tigecycline into treatment regimens was associated with increased success. The overall safety of macrolide-based regimens was moderately satisfactory since no fatalities or disabilities resulted from treatment. However, the total incidence of adverse effects was high. Indeed, there were cases in which patients were unable to tolerate one or more potentially effective drugs, i.e., azithromycin, amikacin, imipenem, linezolid, and tigecycline, during the course of treatment.

Two recent meta-analyses reported disappointing treatment outcomes for *M. abscessus* pulmonary disease. The therapeutic efficiency rates were 54 and 45.6% for all patients, and 35 and 33.0% for patients diagnosed with pulmonary, *M. abscessus* subsp. *abscessus* infections (Pasipanodya et al., 2017; Kwak et al., 2019). Similar rates of treatment success are reported here, i.e., 45.1% for all cases of *M. abscessus* pulmonary disease and 33.5% for cases involving *M. abscessus* subsp. *abscessus*. As such, the therapeutic efficacy of *M. abscessus* pulmonary disease continues to be unsatisfactory, and is even worse for *M. abscessus* subsp. *abscessus* infections.

Amikacin exhibits a high level of antibacterial activity and a low rate of resistance *in vitro*; its successful use to treat pulmonary, *M. abscessus* infections has been reported (Olivier et al., 2014; Lee H. et al., 2017). Indeed, amikacin administered parenterally is regarded as one of the most active antibiotics available to treat *M. abscessus* pulmonary disease (Griffith et al., 2007). Consistent with this perception, amikacin administered in our study was strongly associated with the alleviation of symptoms and treatment success suggesting that amikacin remains an ideal, first choice for treating *M. abscessus* infections. Clinicians should be aware, however, that amikacin is ototoxic. As such, blood concentration of amikacin should be monitored continually to ensure safety.

The anti-*M. abscessus* activity of imipenem *in vitro* is variable; bacterial resistance was over 60% in some studies (Chua et al., 2015; Lee M.C. et al., 2017; Li B. et al., 2017). Imipenem was efficacious, however, in treating pulmonary *M. abscessus* disease in our study. Similar results were reported by Kwak et al. (2019). The elevated antimicrobial activity expressed by imipenem intracellularly provides one plausible explanation for the apparent difference in activity exhibited *in vitro* versus *in vivo* (Rominski et al., 2017). In this regard, the high *in vivo* killing activity of imipenem in an embryonic zebrafish test system was reported (Lefebvre et al., 2016). Moreover, it is likely that the combination of imipenem with other antibiotics has a synergistic or additive effect, which contributes to the treatment success associated with imipenem (Miyasaka et al., 2007; Le Run et al., 2019). Notably, imipenem caused the fewest severe, adverse side effects among the four dominant drugs (i.e., amikacin, imipenem, linezolid, and tigecycline) identified in this study suggesting that it should be included as a treatment option provided *in vitro* sensitivity testing demonstrates the susceptibility of the clinical *M. abscessus* isolate. Furthermore, a newly developed beta-lactamase inhibitor, relebactam, has been shown to significantly improve the anti-*M. abscessus* activity of imipenem *in vitro* and no additional consideration needed to be addressed when imipenem and relebactam are used together (Zhanel et al., 2018; Kaushik et al., 2019a).

Accumulated evidence suggests that linezolid possesses elevated anti-*M. abscessus* activity. Recently, we reported the high activity expressed by linezolid *in vitro* against clinical *M. abscessus* isolates collected from patients with lung diseases (Ye et al., 2019). A study conducted using a *Drosophila melanogaster*-infection model demonstrated the anti-*M. abscessus* activity of linezolid *in vivo* (Oh et al., 2014); the successful use of linezolid in treating clinical *M. abscessus* infections was also reported (Inoue et al., 2018). These results are supported by data presented here. Better outcomes occurred when linezolid was a component of multi-drug therapy used to treat *M. abscessus* pulmonary disease. Linezolid has the advantage that it can be administered orally. It penetrates well into both extracellular fluid and cells, making linezolid one of the more important options for treating *M. abscessus* infections (Honeybourne et al., 2003). Linezolid-induced myelosuppression, however, was the most severe event leading to treatment intervention in our study. Considering its high price and limited availability in some areas, linezolid may be a more appropriate secondary treatment choice, especially when antibiotic sensitivity testing demonstrates alternatives.

Tigecycline exhibits the potentially strongest antibacterial activity of any antibiotic against *M. abscessus in vitro*. One study conducted in Japan showed it exerts 100% bacteriostasis against *M. abscessus* at very low concentrations (MIC ≤ 0.5 μg/ml), which is far superior to the antibacterial effect of clarithromycin (62%) and linezolid (77%) at the CLSI recommended breakpoint (Hatakeyama et al., 2017). Similar results were found in both France (90%, MIC ≤ 1 μg/ml) and China (94.3%, MIC ≤ 2 μg/ml) (Mougari et al., 2016; Li G. et al., 2017). Moreover, the combination of tigecycline with clarithromycin *in vitro* produces synergistic antibacterial effects against *M. abscessus* (Zhang et al., 2017). Tigecycline also showed excellent therapeutic effects against *M. abscessus* infection in a clinical study. Wallace et al. (2014) reported that daily treatment of *M. abscessus* disease with 50–100 mg tigecycline for 1 month resulted in a clinical remission rate that exceeded 60%. Tigecycline also proved superior in treating *M. abscessus* infections in the study reported here, supporting the British Thoracic Society guidelines that list tigecycline as a first-line solution for treating *M. abscessus* infections (Haworth et al., 2017). It is pertinent to note that tigecycline-treated patients often suffered from severe nausea and vomiting. Notably, two newly developed tetracycline analogs, omadacycline and eravacycline, have been reported to show therapeutic potential in treatment of *M. abscessus* infection (Kaushik et al., 2019b; Shoen et al., 2019), with similar *in vitro* activity to tigecycline, but better tolerated.

The study described herein has several limitations. First, it is a retrospective analysis of data obtained at a single center, which could limit the generalization and accuracy of the results. Second, only a relatively small number of *M. abscessus* subsp. *massiliense* infected cases were included, consequently, their characteristics may not be well representative. Third, due to the simultaneous administration of multiple antibiotics, conclusions regarding the adverse effects of individual drugs may be inaccurate. Four, this study excluded subjects who failed to complete their follow-up visits. Conceivably, this failure occurs as a consequence of adverse drug side effects resulting in an underestimation of the adverse events that could otherwise lead to treatment modification. Finally, antibiotics are selected strictly according to guidelines or sputum culture results in our study, rather than at random, resulting in the occurrence of prescription bias. However, it is inevitable.

## Conclusion

The success rate of *M. abscessus* pulmonary disease treatment is still unsatisfactory, albeit the use of amikacin, imipenem, linezolid, and tigecycline is associated with increased treatment success. Adverse effects are common due to the long-term combination anti-*M. abscessus* therapy. Ototoxicity caused by amikacin, gastrointestinal side effects caused primarily by tigecycline, and myelosuppression caused by linezolid were the most severe adverse effects observed.

## Data Availability

The raw data supporting the conclusions of this manuscript will be made available by the authors, without undue reservation, to any qualified researcher.

## Author Contributions

All authors contributed to the preparation of the final manuscript. JC, LZ, YM, BL, and HC conceived and designed the study. MY, QG, YZ, LX, BL, and ZZ collected the clinical data and performed the clinical evaluations. BL and JC performed the statistical analyses. JC, LZ, and YM wrote the manuscript, which was reviewed, edited, and approved by all authors.

## Conflict of Interest Statement

The authors declare that the research was conducted in the absence of any commercial or financial relationships that could be construed as a potential conflict of interest.
