# Supplementary material for: Clinical Efficacy and Adverse Effects of Antibiotics Used to Treat Mycobacterium abscessus Pulmonary Disease
Source: Front Microbiol. 2019 Aug 23;10:1977. doi: 10.3389/fmicb.2019.01977 (PMC6716072; doi:10.3389/fmicb.2019.01977)
Supplement: Supplementary file 1 [file Table_1.DOCX]

Supplementary Table 1. Efficacy of antibiotic treatment: symptomatic improvement^a^

| Antibiotic | *M. abscessus* pulmonary disease  (n=244) | | | | *M. abscessus* subsp. *abscessus* pulmonary disease  (n=185) | | | | *M. abscessus* subsp. *massiliense* pulmonary disease  (n=59) | | | |
| --- | --- | --- | --- | --- | --- | --- | --- | --- | --- | --- | --- | --- |
|  | Total | Improved | Unimproved | *P* value | Total | Improved | Unimproved | *P* value | Total | Improved | Unimproved | *P* value |
| Clarithromycin | 199 | 108 (78.8) | 91 (85.0) | 0.214 | 149 | 65 (74.7) | 84 (85.7) | 0.059 | 50 | 43 (86.0) | 7 (77.8) | 0.615 |
| Azithromycin | 61 | 40 (29.2) | 21 (19.6) | 0.087 | 47 | 30 (34.5) | 17 (17.3) | 0.008 | 14 | 10 (20.0) | 4 (44.4) | 0.195 |
| Amikacin | 218 | 129 (94.2) | 89 (83.2) | 0.006 | 166 | 83 (95.4) | 83 (84.7) | 0.017 | 52 | 46 (92.0) | 6 (66.7) | 0.064 |
| Imipenem | 67 | 44 (32.1) | 23 (21.5) | 0.065 | 47 | 27 (31.0) | 20 (20.1) | 0.097 | 20 | 17 (34.0) | 3 (33.3) | 1.000 |
| Meropenem | 13 | 8 (5.8) | 5 (4.7) | 0.687 | 10 | 6 (6.9) | 4 (4.1) | 0.520 | 3 | 2 (4.0) | 1 (11.1) | 0.397 |
| Cefoxitin | 144 | 81 (59.1) | 63 (58.9) | 0.969 | 110 | 52 (59.8) | 58 (59.2) | 0.935 | 34 | 29 (58.0) | 5 (55.6) | 1.000 |
| Linezolid^b^ | 38 | 25 (18.2) | 13 (12.1) | 0.192 | 27 | 15 (17.2) | 12 (12.2) | 0.337 | 11 | 10 (20.0) | 1 (11.1) | 1.000 |
| Tigecycline | 53 | 38 (27.7) | 15 (14.0) | 0.010 | 39 | 25 (28.7) | 14 (14.3) | 0.016 | 14 | 13 (26.0) | 1 (11.1) | 0.671 |
| Doxycycline | 30 | 15 (10.9) | 15 (14.0) | 0.469 | 23 | 10 (11.5) | 13 (13.3) | 0.716 | 7 | 5 (10.0) | 2 (22.2) | 0.288 |
| Minocycline | 22 | 12 (8.8) | 10 (9.3) | 0.874 | 15 | 6 (6.9) | 9 (9.2) | 0.569 | 7 | 6 (12.0) | 1 (11.1) | 1.000 |
| Moxifloxacin^b^ | 53 | 36 (26.3) | 17 (15.9) | 0.051 | 34 | 21 (24.1) | 13 (13.3) | 0.057 | 19 | 15 (30.0) | 4 (44.4) | 0.450 |
| Levofloxacin^b^ | 26 | 12 (8.8) | 14 (13.1) | 0.277 | 20 | 8 (9.2) | 12 (12.2) | 0.505 | 6 | 4 (8.0) | 2 (22.2) | 0.224 |
| Ciprofloxacin | 17 | 10 (7.3) | 7 (6.5) | 0.818 | 13 | 6 (6.9) | 7 (7.1) | 0.948 | 4 | 4 (8.0) | 0 (0) | 1.000 |

^a^Data are the number (percentage; the number of patients whose symptoms improved or unimproved with the indicated drug divided by the total number of patients whose symptoms improved or unimproved). Each antibiotic listed was included regardless of whether it was discontinued during the course of treatment.

^b^Administered orally and/or intravenously.

Supplementary Table 2. Efficacy of antibiotic treatment: radiographic improvement^a^

| Antibiotic | *M. abscessus* pulmonary disease (n=244) | | | | *M. abscessus* subsp. *abscessus* pulmonary disease (n=185) | | | | *M. abscessus* subsp. *massiliense* pulmonary disease (n=59) | | | |
| --- | --- | --- | --- | --- | --- | --- | --- | --- | --- | --- | --- | --- |
|  | Total | Improved | Unimproved | *P* value | Total | Improved | Unimproved | *P* value | Total | Improved | Unimproved | *P* value |
| Clarithromycin | 199 | 82 (79.6) | 117 (83.0) | 0.503 | 149 | 48 (73.8) | 101 (84.2) | 0.091 | 50 | 34 (89.5) | 16 (76.2) | 0.258 |
| Azithromycin | 61 | 30 (29.1) | 31 (22.0) | 0.203 | 47 | 23 (35.4) | 24 (20.0) | 0.022 | 14 | 7 (18.4) | 7 (33.3) | 0.218 |
| Amikacin | 218 | 96 (93.2) | 122 (86.5) | 0.095 | 166 | 61 (93.8) | 105 (87.5) | 0.175 | 52 | 35 (92.1) | 17 (81.0) | 0.233 |
| Imipenem | 67 | 35 (34.0) | 32 (22.7) | 0.051 | 47 | 21 (32.3) | 26 (21.7) | 0.112 | 20 | 14 (36.8) | 6 (28.6) | 0.521 |
| Meropenem | 13 | 5 (4.9) | 8 (5.7) | 0.778 | 10 | 5 (7.7) | 5 (4.2) | 0.311 | 3 | 0 (0) | 3 (14.3) | 0.041 |
| Cefoxitin | 144 | 63 (61.2) | 81 (57.4) | 0.560 | 110 | 41 (63.1) | 69 (57.5) | 0.461 | 34 | 22 (57.9) | 12 (57.1) | 0.955 |
| Linezolid^b^ | 38 | 23 (22.3) | 15 (10.6) | 0.013 | 27 | 16 (24.6) | 11 (9.2) | 0.004 | 11 | 7 (18.4) | 4 (19.0) | 1.000 |
| Tigecycline | 53 | 31 (30.1) | 22 (15.6) | 0.007 | 39 | 18 (15.0) | 21 (32.3) | 0.006 | 14 | 10 (26.3) | 4 (19.0) | 0.530 |
| Doxycycline | 30 | 8 (7.8) | 22 (15.6) | 0.066 | 23 | 5 (7.7) | 18 (15.0) | 0.150 | 7 | 3 (7.9) | 4 (19.0) | 0.233 |
| Minocycline | 22 | 7 (6.8) | 15 (10.6) | 0.301 | 15 | 4 (6.2) | 11 (9.2) | 0.474 | 7 | 3 (7.9) | 4 (19.0) | 0.205 |
| Moxifloxacin^b^ | 53 | 26 (25.2) | 27 (19.1) | 0.254 | 34 | 14 (21.5) | 20 (16.7) | 0.414 | 19 | 12 (31.6) | 7 (33.3) | 0.890 |
| Levofloxacin^b^ | 26 | 8 (7.8) | 18 (12.8) | 0.211 | 20 | 5 (7.7) | 15 (12.5) | 0.315 | 6 | 3 (7.9) | 3 (14.3) | 0.656 |
| Ciprofloxacin | 17 | 7 (6.8) | 10 (7.1) | 0.929 | 13 | 3 (4.6) | 10 (8.3) | 0.548 | 4 | 4 (10.5) | 0 (0) | 0.286 |

^a^ Data are the number (percentage; the number of patients who demonstrated radiographic improvement or not with the indicated drug divided by the total number of patients who demonstrated radiographic improvement or not). Each antibiotic listed was included regardless of whether it was discontinued during the course of treatment.

^b^Administered orally and/or intravenously.

Supplementary Table 3. Correlation between treatment with a single antibiotic and symptomatic improvement

| Antibiotic | Total  (n=244) | | | *M. abscessus* subsp. *abscessus* pulmonary disease (n=185) | | | *M. abscessus* subsp. *massiliense* pulmonary disease (n=59) | | |
| --- | --- | --- | --- | --- | --- | --- | --- | --- | --- |
|  | Adjusted OR^a,b^ | 95% CI^a^ | *P* value | Adjusted OR | 95% CI | *P* value | Adjusted OR | 95% CI | *P* value |
| Clarithromycin | 0.620 | 0.308-1.248 | 0.180 | 0.501 | 0.231-1.087 | 0.080 | 1.486 | 0.217-10.198 | 0.687 |
| Azithromycin | 1.730 | 0.933-3.207 | 0.082 | 2.507 | 1.237-5.083 | 0.011 | 0.292 | 0.060-1.405 | 0.124 |
| Amikacin | 3.412 | 1.389-8.383 | 0.007 | 4.344 | 1.326-14.225 | 0.015 | 17.027 | 1.379-210.246 | 0.027 |
| Imipenem | 1.704 | 0.945-3.073 | 0.076 | 1.682 | 0.853-3.317 | 0.133 | 1.010 | 0.216-4.711 | 0.990 |
| Meropenem | 1.218 | 0.390-3.806 | 0.735 | 1.787 | 0.486-6.574 | 0.382 | 0.341 | 0.026-4.487 | 0.413 |
| Cefoxitin | 1.103 | 0.650-1.872 | 0.717 | 1.109 | 0.600-2.048 | 0.741 | 1.062 | 0.248-4.556 | 0.935 |
| Linezolid^c^ | 1.510 | 0.728-3.134 | 0.268 | 1.422 | 0.614-3.291 | 0.411 | 2.198 | 0.237-20.344 | 0.488 |
| Tigecycline | 2.040 | 1.079-3.857 | 0.028 | 1.971 | 0.931-4.173 | 0.076 | 2.614 | 0.291-23.514 | 0.391 |
| Doxycycline | 0.599 | 0.260-1.380 | 0.229 | 0.628 | 0.222-1.772 | 0.379 | 0.408 | 0.053-3.147 | 0.390 |
| Minocycline | 0.992 | 0.399-2.467 | 0.986 | 0.691 | 0.206-2.315 | 0.549 | 1.312 | 0.116-14.876 | 0.827 |
| Moxifloxacin^c^ | 1.438 | 0.769-2.689 | 0.255 | 1.155 | 0.524-2.546 | 0.720 | 0.669 | 0.135-3.305 | 0.622 |
| Levofloxacin^c^ | 0.474 | 0.193-1.162 | 0.103 | 0.453 | 0.142-1.445 | 0.181 | 0.242 | 0.032-1.857 | 0.172 |
| Ciprofloxacin | 0.975 | 0.353-2.689 | 0.960 | 0.866 | 0.248-3.029 | 0.822 | 0 | 0 | 0 |

^a^Abbreviations: OR, odds ratio; CI, confidence interval.

^b^Adjusted for age, sex, body mass index and radiographic findings.

^c^Administered orally and/or intravenously.

Supplementary Table 4. Correlation between treatment with a single antibiotic and radiographic improvement

| Antibiotic | Total  (n=244) | | | *M. abscessus* subsp. *abscessus* pulmonary disease (n=185) | | | *M. abscessus* subsp. *massiliense* pulmonary disease (n=59) | | |
| --- | --- | --- | --- | --- | --- | --- | --- | --- | --- |
|  | Adjusted OR^a,b^ | 95% CI^a^ | *P* value | Adjusted OR | 95% CI | *P* value | Adjusted OR | 95% CI | *P* value |
| Clarithromycin | 0.744 | 0.369-1.499 | 0.408 | 0.545 | 0.246-1.206 | 0.134 | 1.694 | 0.320-8.968 | 0.535 |
| Azithromycin | 1.509 | 0.819-2.780 | 0.187 | 2.111 | 1.034-4.308 | 0.040 | 0.522 | 0.133-2.053 | 0.353 |
| Amikacin | 2.254 | 0.877-5.790 | 0.091 | 2.454 | 0.738-8.159 | 0.143 | 3.889 | 0.601-25.147 | 0.154 |
| Imipenem | 1.735 | 0.968-3.109 | 0.064 | 1.646 | 0.821-3.301 | 0.161 | 1.362 | 0.387-4.789 | 0.630 |
| Meropenem | 0.715 | 0.223-2.294 | 0.573 | 1.657 | 0.451-6.093 | 0.447 | 0 | 0 | 0 |
| Cefoxitin | 1.306 | 0.765-2.231 | 0.328 | 1.433 | 0.753-2.725 | 0.273 | 1.001 | 0.306-3.272 | 0.998 |
| Linezolid^c^ | 2.250 | 1.096-4.618 | 0.027 | 3.142 | 1.328-7.429 | 0.009 | 1.093 | 0.238-5.025 | 0.909 |
| tigecycline | 2.141 | 1.136-4.034 | 0.019 | 2.319 | 1.099-4.891 | 0.027 | 1.131 | 0.295-4.334 | 0.857 |
| Doxycycline | 0.472 | 0.197-1.135 | 0.093 | 0.457 | 0.154-1.355 | 0.158 | 0.646 | 0.094-4.453 | 0.657 |
| Minocycline | 0.588 | 0.226-1.534 | 0.278 | 0.642 | 0.192-2.149 | 0.473 | 0.229 | 0.037-1.420 | 0.113 |
| Moxifloxacin^c^ | 1.373 | 0.735-2.564 | 0.319 | 1.240 | 0.568-2.706 | 0.590 | 0.946 | 0.257-3.483 | 0.933 |
| Levofloxacin^c^ | 0.540 | 0.221-1.324 | 0.178 | 0.566 | 0.192-1.666 | 0.301 | 0.343 | 0.057-2.063 | 0.242 |
| Ciprofloxacin | 0.875 | 0.313-2.446 | 0.799 | 0.521 | 0.135-2.012 | 0.344 | 0 | 0 | 0 |

^a^Abbreviations: OR, odds ratio; CI, confidence interval.

^b^Adjusted for age, sex, body mass index and radiographic findings.

^c^Administered orally and/or intravenously.
